# Cerebral/Cortical Visual Impairment in Primary Care: Extending the Evidence to Family Medicine

**DOI:** 10.7759/cureus.116846

**Published:** 2026-09-24

**Authors:** John D Rogers, Harlan Sayles, Richard H Legge

**Affiliations:** 1 Department of Ophthalmology, University of Nebraska Medical Center, Omaha, USA; 2 College of Public Health, University of Nebraska Medical Center, Omaha, USA

**Keywords:** cerebral/cortical visual impairment, family medicine physicians, referral practices, survey research, vision screening

## Abstract

Introduction

Cerebral/cortical visual impairment (CVI) is a leading cause of childhood visual impairment but remains underrecognized. CVI-related education, awareness, and visual dysfunction screening practices among family medicine physicians remain poorly characterized. This study evaluated CVI education and awareness, visual dysfunction screening, and referral practices among family medicine physicians. This present study evaluated whether similar patterns extend to family medicine physicians.

Methods

Nebraska family medicine physicians completed an anonymous 60-question electronic survey assessing CVI education and awareness, screening for visual dysfunction in children with neurologic disease, referral practices, and intended use of hypothetical screening tools.

Results

Fifty-seven responses were analyzed, with varying response numbers for each question. Fifty-four (98.2%) respondents reported no CVI education during residency/fellowship, and 51 (92.7%) reported no CVI continuing medical education. Forty-three (78.2%) respondents also expressed interest in future CVI education. Fifty (92.6%) were unaware of CVI diagnostic criteria, and 38 (70.4%) respondents reported screening for visual dysfunction in children with neurologic disease in 25% of encounters or less. Intended use was greater for shorter hypothetical screening approaches. Referral pathways and reported specialist availability varied.

Conclusions

Family medicine physicians reported limited CVI education and infrequent visual dysfunction screening. These findings suggest that opportunities to improve CVI education and recognition may extend across primary care specialties. This study also underscores potential opportunities for future efforts to improve recognition of children who may warrant further evaluation for CVI.

## Introduction

Cerebral/cortical visual impairment (CVI) is a leading cause of childhood visual impairment in developed countries and remains frequently underrecognized [1,2]. Unlike visual impairment resulting primarily from ocular pathology, CVI arises from abnormalities affecting the brain and its processing of visual information [1,2]. Clinical manifestations are heterogeneous and may include difficulties with visual function despite relatively preserved visual acuity [1,2]. CVI also frequently occurs in children with neurologic conditions [1], making recognition of visual dysfunction particularly important in this population. Delayed recognition may prevent affected children from receiving timely evaluation and appropriate educational and supportive services [1,3].

Primary care clinicians are positioned to play a role in recognizing visual concerns during childhood. Professional recommendations include family physicians among clinicians responsible for age-appropriate pediatric vision screening [4,5]. Current screening practices, however, are primarily directed toward detecting ocular abnormalities, amblyopia, and amblyopia risk factors, while specific guidance for identifying CVI in primary care remains limited [1,5-7]. This distinction may be important because the functional visual difficulties associated with CVI may not be adequately captured through conventional measures of visual acuity alone [1,2]. Although questionnaires intended to identify children who may warrant evaluation for CVI have been developed, limitations in validation and implementation have restricted their incorporation into routine clinical practice [3,8]. 

Although family physicians participate in pediatric vision assessment, CVI-specific education, awareness, screening practices, and referral patterns have not been well characterized in this population. We previously examined CVI-related education and clinical practices among pediatricians in Nebraska and identified limited familiarity with CVI and infrequent reported screening for visual dysfunction among children with neurologic disease [9]. Whether similar patterns are present among other primary care clinicians is unknown. Family physicians represent an important population to examine because they provide preventive and longitudinal care to children across a variety of practice environments, including communities where pediatric or ophthalmic specialty services may be less readily accessible [4,5]. Characterizing their familiarity with CVI, current approaches to visual dysfunction, and referral practices may therefore provide a broader understanding of opportunities for CVI recognition within primary care.

The present study evaluated CVI education and awareness, visual dysfunction screening, and referral practices among family medicine physicians in Nebraska. We also assessed intended use of hypothetical screening approaches with varying administration requirements. By extending our previous investigation to a separate primary care cohort, this study sought to determine whether previously observed gaps in CVI familiarity and visual dysfunction screening were also present among family physicians and to identify considerations for future educational and screening initiatives in primary care.

## Materials and methods

Survey instrument and procedure

A 60-item survey adapted from the instrument previously described by Rogers et al. [9] was used in this study. The original instrument was developed by a board-certified ophthalmologist in collaboration with a survey-methodology expert. For the present study, practice-setting response options were modified to reflect family medicine practice environments. These modifications were reviewed by the same ophthalmologist and survey-methodology expert before distribution. The electronic survey was also tested before distribution to confirm that it functioned as intended. Although the instrument underwent expert review and functional testing, it did not undergo formal assessment of content or face validity or psychometric validation.

For the present study, the same survey framework and clinical questions were retained, with practice-setting response options modified to reflect family medicine practice environments. These response options included “Family Medicine - MD clinic based,” “Family Medicine - PA clinic based,” “Family Medicine - RNP clinic based,” “Family Medicine - MD, PA, or RNP hospital based,” and “Family Medicine - MD, PA, or RNP emergency department based.” The full survey is provided in the Appendices. Questions 58-60 of the previously described survey were excluded from the present analysis.

The survey was administered using REDCap (Vanderbilt University, Nashville, USA) [10,11], following the general distribution procedures described by Rogers et al. [9]. Participants were provided with information regarding the purpose of the study, potential risks, and study contact information. Participation was voluntary and anonymous, and no incentives were offered. The study was reviewed by the Institutional Review Board at our institution, University of Nebraska Medical Center, and determined to be exempt from full review (IRB #0723-24-EX). Informed consent was obtained through survey completion, with participants informed that completion of the survey constituted consent to participate.

Family medicine physicians practicing in Nebraska were contacted by email using a distribution list provided through the Nebraska Healthcare Workforce Office. The investigators had access to the distribution mechanism but not to the underlying recipient list or delivery records. Consequently, the total number of email addresses included in the distribution, the number of successfully delivered invitations, the number of undeliverable messages, and the number of eligible actively practicing family medicine physicians who received an invitation were unavailable to the study team. Therefore, a conventional survey response rate could not be calculated. Data were collected from May through December 2025, with reminder emails distributed approximately every two weeks.

Participants

An email list of family medicine physicians in Nebraska was obtained from the Nebraska Healthcare Workforce Office. Licensed family medicine physicians practicing in Nebraska who accessed the distributed survey and completed at least one survey item were eligible for inclusion. A total of 58 responses were received. One respondent did not proceed beyond the second survey question and was excluded, leaving 57 responses for analysis.

Measured variables

Measured variables were similar to those evaluated in Rogers et al. [9] and were grouped into four domains: general practice characteristics, CVI education, visual dysfunction screening practices, and referral practices.

Statistical analysis

Survey responses were analyzed descriptively. REDCap was used for data collection and management, and Microsoft Excel (Microsoft Corporation, Redmond, WA) was used for statistical analysis. Categorical variables were summarized using frequencies and percentages. No inferential statistical analyses were performed. ZIP codes were compared to identify obvious duplicates; none were found. Because responses were anonymous and ZIP codes are not unique identifiers, duplicate participation could not be definitively excluded.

Because individual survey items were optional, partially completed surveys were retained in the analysis. Each item was analyzed using all available responses, with missing responses excluded only from the analysis of the corresponding item. Accordingly, denominators varied across survey questions and reflect the number of respondents who answered each individual item. Item-specific denominators are reported as n/N (%) in the corresponding tables.

## Results

General information

Respondent characteristics are summarized in Table 1. Forty-five (81.9%) respondents reported having more than 10 years of experience, 48 (87.3%) reported working full-time, and 46 (83.6%) reported practicing in non-academic settings. Among those practicing in non-academic settings, 23 (50.0%) reported being greater than a 1-hour drive from the Omaha or Lincoln metropolitan areas. Sixteen (34.8%) reported being in a large metropolitan area, and seven (15.2%) reported being within 1 hour of a large metropolitan area. 

**Table 1:** Respondent characteristics and general practice information MD: Doctor of Medicine; PA: Physician Assistant; RNP: Registered Nurse Practitioner

Characteristic	Value
Clinic Description	n/N (%)
Family Medicine - MD clinic based	49/57 (86.0%)
Family Medicine - PA clinic based	0/57 (0%)
Family Medicine - RNP clinic based	0/57 (0%)
Family Medicine - MD, PA or RNP Hospital based	3/57 (5.3%)
Family Medicine - MD, PA, or RNP Emergency Department based	5/57 (8.8%)
Years in Practice	n/N (%)
>20 years	25/55 (45.5%)
11-20 Years	20/55 (36.4%)
3-10 years	6/55 (10.9%)
<3 years	4/55 (7.3%)
Employment Status	n/N (%)
Full time	48/55 (87.3%)
Part time	7/55 (12.7%)
Practice Setting	n/N (%)
Academic	9/55 (16.4%)
Non-academic	46/55 (83.6%)

Education on CVI

Respondents reported limited previous education regarding CVI (Table 2). Fifty-four (98.2%) respondents reported not receiving CVI education during residency or fellowship, and 51 (92.7%) reported not receiving CVI education through continuing medical education. Despite this, 43 (78.2%) respondents reported being somewhat or very likely to attend CVI-focused continuing medical education within the next two years. Among the 12 respondents who were somewhat or very unlikely to attend, five (41.7%) identified CVI as a low-priority topic. Respondents also reported limited familiarity with CVI terminology. Most respondents reported being unfamiliar with commonly used CVI terminology, and 50 (92.6%) reported being unaware of diagnostic criteria for CVI. None of the 54 respondents answering the question reported being able to independently diagnose CVI.

**Table 2:** Respondent CVI education and awareness CVI: cerebral/cortical visual impairment; CME: Continuing Medical Education

Characteristic	Value
CVI education and diagnostic familiarity	n/N (%)
No CVI lecture/conference during residency or fellowship	54/55 (98.2%)
No CVI lecture during continuing medical education	51/55 (92.7%)
Somewhat/very likely to attend CVI CME within 2 years	43/55 (78.2%)
Unaware of criteria used to diagnose CVI	50/54 (92.6%)
Unable to make a diagnosis of CVI	54/54 (100.0%)
Awareness of CVI terminology	n/N (%)
Had not heard the term “CVI”	39/53 (73.6%)
Had not heard “cortical vision impairment”	39/53 (73.6%)
Had not heard “cerebral vision impairment”	40/53 (75.5%)
Had not heard “brain-based vision impairment”	37/53 (69.8%)
Did not know what CVI stands for	42/53 (79.2%)
Had not heard of the Pediatric Cortical Visual Impairment Society	52/53 (98.1%)

Visual dysfunction screening

Thirty-seven of 54 respondents (68.5%) reported screening for visual dysfunction in children with neurologic disease. In a separate item presented to all respondents regardless of their response to the preceding screening question, 38 of 54 (70.4%) reported screening for visual dysfunction in 25% or fewer of encounters with children with neurologic disease. Other visual dysfunction screening results are listed in Table 3.

**Table 3:** Respondent CVI screening practices CVI: cerebral/cortical visual impairment

Characteristic	Value
Current Screening Practices	n/N (%)
Question parents about visual dysfunction	32/37 (86.5%)
Assess visual dysfunction through physical examination	33/37 (89.2%)
Use a vision screening device	9/37 (24.3%)
Intended use of hypothetical screening tools	n/N (%)
≤3 questions, ≤2 min; would use in ≥75% of encounters	31/54 (57.4%)
≤2 questions + 1 physical examination, ≤2 min; would use in ≥75% of encounters	30/54 (55.6%)
≤5 questions, ≤4 min; would use in ≥75% of encounters	16/54 (29.6%)
≤10 questions, ≤8 min; would use in ≥75% of encounters	7/54 (13.0%)
Somewhat/highly likely to use free mobile screening tool requiring ≤5 min	51/54 (94.4%)

Referral practices

Pediatric ophthalmology was the most frequently selected specialty for referral following a positive actual or hypothetical visual dysfunction screening result. This was followed by neuro-ophthalmology and general ophthalmology, pediatric neuro-ophthalmology, and pediatric neurology. Additional findings regarding referral preferences and accessibility are presented in Table 4.

**Table 4:** Respondent CVI referral practices CVI: cerebral/cortical visual impairment; NA: Not Applicable

Specialist Receiving Referral	Number of Respondents Referring to Specialist, n/N (%)	Number Reporting That the Referred Specialist Is in Their Geographic Area, n/N (%)	Number Reporting That the Specialist Is Easily Contacted if in Geographic Area, n/N (%)
Neurologist	14/54 (25.9%)	11/14 (78.6%)	9/11 (81.8%)
Neuro-Ophthalmologist	28/54 (51.9%)	21/28 (75.0%)	18/21 (85.7%)
Neuro-Optometrist	4/54 (7.4%)	2/4 (50.0%)	2/2 (100.0%)
Occupational Therapist	4/54 (7.4%)	4/4 (100.0%)	2/4 (50.0%)
Ophthalmologist	28/54 (51.9%)	27/28 (96.4%)	27/27 (100.0%)
Optometrist	10/54 (18.5%)	10/10 (100.0%)	10/10 (100.0%)
Pediatric Neurologist	20/54 (37.0%)	11/20 (55.0%)	10/11 (90.9%)
Pediatric Neuro-Ophthalmologist	27/54 (50.0%)	9/27 (33.3%)	7/9 (77.8%)
Pediatric Ophthalmologist	29/54 (53.7%)	18/29 (62.1%)	18/18 (100.0%)
Pediatric Optometrist	8/54 (14.8%)	1/8 (12.5%)	1/1 (100.0%)
Physiatrist	0/54 (0%)	NA	NA
Physical Therapist	2/54 (3.7%)	2/2 (100.0%)	1/2 (50.0%)
Vision Teacher	2/54 (3.7%)	1/2 (50.0%)	1/1 (100.0%)
None	0/54 (0%)	NA	NA

## Discussion

This study extends our previous investigation of CVI-related education and clinical practices among pediatricians by examining a separate cohort of family medicine physicians in Nebraska [9]. Similar to the pediatrician cohort, family medicine respondents reported limited previous education regarding CVI, infrequent screening for visual dysfunction in children with neurologic disease, and substantial interest in additional CVI education and brief approaches to screening. The consistency of several findings across two primary care populations suggests that limited familiarity with CVI may not be restricted to a single specialty. At the same time, differences in reported awareness and screening practices between the cohorts may reflect their distinct roles in pediatric care.

Limited familiarity with CVI was evident in both physician populations. In the present study, 50 (92.6%) family medicine respondents reported being unaware of criteria used to diagnose CVI, similar to the 76 (96.2%) previously observed among pediatricians [9]. However, 42 (79.2%) family medicine respondents did not know what the abbreviation CVI represented, compared with 43 (55.1%) pediatrician respondents in the previous study. These findings should be interpreted descriptively because the studies were not designed to directly compare the two physician populations. Nevertheless, the high proportion of respondents in both groups reporting limited familiarity with CVI diagnostic criteria is consistent with broader reports of gaps in CVI education among health care professionals [9]. The limited previous educational exposure reported by family physicians may represent one contributor to this unfamiliarity, although the present study was not designed to establish such a relationship. The limited familiarity with CVI diagnostic criteria is particularly relevant given ongoing efforts to establish multidisciplinary approaches to CVI diagnosis and referral [12].

The frequency of visual dysfunction screening also provides an important point of comparison. Among family medicine respondents, 38 (70.4%) reported screening for visual dysfunction in 25% or fewer of encounters with children with neurologic disease. In our previous pediatrician cohort, this number was 32 (40.5%) [9]. Although these questions assessed visual dysfunction rather than CVI-specific screening, the finding is relevant because children with neurologic disease are at increased risk for CVI [1]. Family physicians, like pediatricians, participate in childhood vision assessment [4,5], although the frequency with which they encounter children with complex neurologic disease may differ. Consequently, differences in reported screening between the cohorts should not necessarily be interpreted as differences in quality of care. Instead, the findings demonstrate that opportunities to recognize visual dysfunction in children at increased risk for CVI may exist across multiple primary care settings, especially given that previous studies of both family physicians and pediatricians have similarly demonstrated variability in pediatric vision screening practices and identified practical barriers to screening [4,13].

Despite limited familiarity with CVI, interest in additional education was remarkably similar between the two cohorts. In the present study, 43 (78.2%) family medicine respondents reported being somewhat or very likely to attend CVI-focused continuing medical education within the next two years, compared with 64 (77.1%) pediatrician respondents in our previous study [9]. This consistency demonstrates similar self-reported interest in CVI-focused continuing education among respondents in both cohorts. For family physicians, such education may be most useful if focused on recognizing children at increased risk for CVI, identifying concerns regarding functional vision, and determining when specialty evaluation is warranted rather than on independently establishing a CVI diagnosis.

Preferences regarding hypothetical screening approaches also showed similarities to our previous findings. Family medicine respondents reported greater intended use of shorter hypothetical screening tools than longer tools, consistent with the pattern previously observed among pediatricians [9]. More than half of family medicine respondents indicated that they would use a tool requiring two minutes or less in at least 75% of applicable encounters, while intended use decreased as the proposed administration time increased (Table 3). Additionally, 51 (96.2%) reported being somewhat or highly likely to use the hypothetical free mobile screening tool described in the survey, compared with 69 (87.3%) pediatrician respondents. Questionnaire-based approaches have previously demonstrated potential utility in identifying children who warrant further CVI evaluation [8,14], and these findings support the importance of considering time and clinical workflow when developing potential screening approaches for primary care. However, the hypothetical scenarios differed in characteristics and response formats, preventing conclusions regarding which specific features influenced intended use. Furthermore, intended use should not be interpreted as evidence of actual clinical adoption. Stated willingness to use a hypothetical screening tool should not be interpreted as evidence of real-world adoption. Of note, implementation would additionally depend on factors not assessed in this study, including tool validation, workflow integration, training requirements, and availability of appropriate referral pathways.

Referral patterns provide another area in which the family medicine findings add context to our previous work. Pediatric ophthalmology was the most commonly selected referral destination among family medicine respondents, consistent with our previous pediatrician cohort, although both groups reported referrals across multiple ophthalmic and neurologic specialties [9,15]. Challenges surrounding CVI referral have also been reported among other healthcare professionals, including perceived lack of clarity in referral pathways [16]. Importantly, half of family medicine respondents practicing in non-academic environments were located more than a one-hour drive from the Omaha or Lincoln metropolitan areas. This geographic distribution raises additional questions regarding access to specialty evaluation, although distance alone cannot establish the presence of an access barrier. Prior studies have identified additional barriers to specialty follow-up after abnormal childhood vision screening [17]. Future studies should evaluate referral completion, appointment availability, travel burden, and other factors affecting access to CVI-related specialty care.

Taken together with our previous pediatrician study [9], these findings suggest that gaps in CVI education and familiarity are present across more than one group of primary care physicians in Nebraska. The similarity in interest in continuing education and preferences for brief hypothetical screening approaches also suggests potential opportunities for initiatives that extend across primary care specialties rather than targeting only pediatricians. Future studies incorporating larger and more geographically diverse physician populations could determine whether these patterns persist outside Nebraska and directly evaluate differences between specialties. Prospective studies would also be necessary to determine whether education or validated screening strategies change actual recognition and referral practices.

This study has several limitations. The number of eligible physicians who received the survey invitation could not be determined, preventing calculation of a conventional response rate. With only 57 analyzed responses, substantial non-response bias is possible, and the respondents may not be representative of family medicine physicians practicing throughout Nebraska. Participation was voluntary, introducing additional self-selection bias. Also, the survey instrument was not formally validated, and the family medicine adaptation did not undergo separate formal assessment of content or face validity or pilot testing among family medicine physicians. Although the instrument was adapted from a previously developed survey that incorporated clinical and survey-methodology input, these limitations may affect the interpretation and reproducibility of individual survey items. Screening and referral practices were self-reported and may differ from actual clinical behavior, while questions concerning proposed screening tools measured intended rather than observed use. Optional items resulted in varying denominators across analyses. Several questions assessed visual dysfunction broadly rather than CVI-specific screening. Finally, comparisons with our previously studied pediatrician cohort [9] are descriptive and should be interpreted cautiously because the studies were not designed or powered for direct between-group comparisons.

## Conclusions

Family medicine physicians in this Nebraska sample reported limited previous education and familiarity with CVI, similar to patterns previously observed in a separate sample of Nebraska pediatricians. Respondents also reported infrequent screening for visual dysfunction among children with neurologic disease and substantial interest in additional CVI education and brief hypothetical screening approaches. Together, these studies identify similar patterns among two physician samples in Nebraska. Larger, geographically diverse studies are needed to determine whether these findings extend to broader primary care populations and whether educational or screening strategies influence clinical practice.

## Appendices

Example of the survey distributed to respondents.

Question 1: Do you see children with neurologic disease?

A.       Yes

B.       No

Question 2: Your primary office zip code:

                  (Free responses were collected)

Question 3: Which of the following best describes your practice? Choose one.

A.       Family Medicine - MD clinic based

B.       Family Medicine - PA clinic based

C.       Family Medicine - RNP clinic based

D.       Family Medicine - MD, PA or RNP Hospital Based

E.       Family Medicine - MD, PA, or RNP Emergency Department Based

F.       Other, Please Specify

Question 4: How many years have you been in practice since completing your last year of post-graduate training, such as your residency or fellowship? Choose one.

A.       0-2

B.       3-10

C.      11-20

D.      >20

Question 5: Do you consider yourself a full-time or part-time practitioner when you consider your time taking care of patients? Choose one.

A.       Full-time

B.       Part-time

Question 6: Would you describe your practice as primarily academic or non-academic? Choose one.

A.       Academic

B.       Non-academic

Question 7: If academic, which institution is your primary affiliation? Choose one.

A.       Boys Town National Research Hospital/Creighton University

B.       Children's/UNMC

C.       Nebraska Medicine/UNMC

D.       Other, Please list

E.       None

*If “Non-academic” was selected, respondents were directed to question 8.

Question 8: If non-academic, where is the majority of your practice time spent?

F.       Omaha metropolitan area or contiguous suburbs such as Bellevue

G.      Lincoln metropolitan area

H.      Within a 1-hour drive of the city limits of either the Omaha or Lincoln metropolitan areas

I.        Greater than a 1-hour drive from the Omaha or Lincoln metropolitan areas

Question 9: During your residency or fellowship, did you ever attend a lecture or conference on the topic of CVI?

A.       Yes

B.       No

Question 10: In your continuing medical education experience since leaving training, have you ever attended a lecture on CVI?

A.       Yes

B.       No

Question 11: If there were continuing medical education courses available at your local or national professional meetings, either live or virtual, how likely or unlikely is it that you would attend at least one in a 2-year period?

A.       Very likely

B.       Somewhat likely

C.       Somewhat unlikely

D.       Very unlikely

*If “Somewhat unlikely” or “Very unlikely” was selected, respondents were directed to question 12.

Question 12: If you answered "Somewhat unlikely" or "Very unlikely", why would that be? Choose all that apply.

A.       CVI is a low priority subject

B.       Lack of Time

C.       CVI is too complex

D.       CVI is too obscure

E.       Other

Question 13: Are you aware of diagnostic criteria that are used to diagnose CVI?

A.       Yes

B.       No

Question 14: Are you able to make the diagnosis of CVI?

A.       Yes

B.       No

Question 15: In children with neurologic disease, do you screen for visual dysfunction by any method?

A.       Yes

B.       No

*If “yes” was selected, respondents were directed to Question 16.

Question 16: If you answered "Yes", which method do you use to screen for CVI?  Check all that apply.

A.       Questioning Parents

B.       Physical Exam

C.       Other, Please describe (Free responses collected as necessary)

Question 17: If you screen for visual dysfunction in children with neurologic disease, please estimate the percentage out of all your patient encounters where you would do such screening. Choose one.

A.       Never

B.       About 1-10% of the time

C.       About 11-25% of the time

D.       About 26-74% of the time

E.       About 75-89% of the time

F.       About 90-100% of the time

Question 18: If you had a screening tool for visual dysfunction in children with neurologic disease consisting of no more than 3 questions that would take no more than 2 minutes to administer, please estimate how often you would use it. Choose one.

A.       Never

B.       About 1-10% of the time

C.       About 11-25% of the time

D.       About 26-74% of the time

E.       About 75-89% of the time

F.       About 90-100% of the time

Question 19: If you had a screening tool for visual dysfunction in children with neurologic disease consisting of no more than 2 questions and one physical exam test, that would take no more than 2 minutes to administer, please estimate how often you would use it. Choose one.

A.       Never

B.       About 1-10% of the time

C.       About 11-25% of the time

D.       About 26-74% of the time

E.       About 75-89% of the time

F.       About 90-100% of the time

Question 20: If you had a screening tool for visual dysfunction in children with neurologic disease consisting of no more than 5 questions that would take no more than 4 minutes to administer, please estimate how often you would use it. Choose one.

A.       Never

B.       About 1-10% of the time

C.       About 11-25% of the time

D.       About 26-74% of the time

E.       About 75-89% of the time

F.       About 90-100% of the time

Question 21: If you had a screening tool for visual dysfunction in children with neurologic disease consisting of no more than 10 questions that would take no more than 8 minutes to administer, please estimate how often you would use it. Choose one.

A.       Never

B.       About 1-10% of the time

C.       About 11-25% of the time

D.       About 26-74% of the time

E.       About 75-89% of the time

F.       About 90-100% of the time

Question 22: If there was a highly sensitive electronic screening tool for the detection of CVI using a free App on a cell phone or tablet device, and it would take no more than 5 minutes to administer, how likely would you be to use it?

A.       Highly likely

B.       Somewhat likely

C.       Not likely

Question 23: If you were using a screening tool of any kind, either hypothetically or actually, and the child would test "positive", would you refer the patient to any of the following specialists? Choose all that apply.

A.       None

B.       Neurologist

C.       Neuro-Ophthalmologist

D.       Neuro-Optometrist

E.       Occupational Therapist

F.       Ophthalmologist

G.       Optometrist

H.       Pediatric Neurologist

I.         Pediatric Neuro-Ophthalmologist

J.        Pediatric Ophthalmologist

K.        Pediatric Optometrist

L.        Physiatrist

M.       Physical Therapist

N.       Vision Teacher

O.       Other, Please specify

Questions 24-51 concerned the respondents’ answers to question 23. For each respective answer choice, the following question was asked:

For selection "[Answer from Question 23]", are such practitioners in your typical geographic referral area?

A.       Yes

B.       No

*If “yes” was selected for the above question, the following question was asked:

Can you easily obtain this practitioner’s name and contact information?

A.       Yes

B.       No

Question 52: Prior to this survey, have you heard the term "CVI"?

A.       Yes

B.       No

Question 53: Have you heard the term "Cortical Vision Impairment", other than when it was used in this survey?

A.       Yes

B.       No

Question 54: Have you heard the term "Cerebral Vision Impairment", other than when it was used in this survey?

A.       Yes

B.       No

Question 55: Have you heard the term "Brain-based Vision Impairment", other than when it was used in this survey?

A.       Yes

B.       No

Question 56: Prior to this survey, did you know what "CVI" stands for?

A.       Yes

B.       No

Question 57: Have you heard of the Pediatric Cortical Visual Impairment Society, www.pcvis.vision?

A.       Yes

B.       No

Question 58: If you have heard of the Pediatric Cortical Visual Impairment Society, did you know that you can refer patients to its website for information on CVI?

A.       Yes

B.       No

Question 59: Have you ever referred a patient to the Pediatric Cortical Visual Impairment Society website?

A.       Yes

B.       No

Question 60: Screening for vision impairment can be done with a child by having the child make eye contact with you at 1-3 feet of distance, then slowly move your head to see if he/she maintains eye contact. What is the earliest age you would feel comfortable conducting this vision screening technique?

A.       6 months

B.       1 year

C.      18 months

D.       2 years

E.        I don't think I could make this assessment.
